# Cu Isotopic Composition in Surface Environments and in Biological Systems: A Critical Review

**DOI:** 10.3390/ijerph14050538

**Published:** 2017-05-18

**Authors:** Zhuhong Wang, Jiubin Chen, Ting Zhang

**Affiliations:** 1 State Key Laboratory of Environmental Geochemistry, Institute of Geochemistry, Chinese Academy of Sciences, Guiyang 550081, China; wangzhuhong@mail.gyig.ac.cn; 2 Institute of Surface-Earth System Science Research, Tianjin University, Tianjin 300072, China; zhangting_cug@163.com

**Keywords:** Cu isotopic composition, environment, biological systems

## Abstract

Copper (Cu) is a transition metal and an essential micronutrient for organisms, but also one of the most widespread toxic inorganic contaminants at very high content. The research on Cu isotopes has grown rapidly in the last decade. Hitherto, a large number of studies have been published on the theoretical fractionation mechanisms, experimental data and natural variations of Cu isotopes in variable environments and ecosystems. These studies reported a large variation of δ^65^Cu (−16.49 to +20.04‰) in terrestrial samples and showed that Cu isotopes could be fractionated by various biogeochemical processes to different extent. Several papers have previously reviewed the coupling of Cu and Zn isotope systematics, and we give here a tentative review of the recent publications only on Cu isotopesin variable surface repositories, animals and human beings, with a goal to attract much attention to research on Cu (and other metals) behaviors in the environment and biological systems.

## 1. Introduction

Copper (Cu) is a transition metal and a ubiquitous biolimiting micronutrient that involves in many enzymes and regulatory proteins as a cofactor in almost all living organisms [1]. On the other hand, it is also one of the most widespread toxic inorganic contaminants at very high content [2], thus once released into the environment, it potentially threatens ecosystems and the biota (and even human beings) [3]. Cu has two stable isotopes, ^65^Cu (69.17%) and ^63^Cu (30.83%). Cu isotopes are proven to be helpful for understanding sources of Cu and processes that Cu undergoes in surface environments. Cu isotope studies have an apparently longer history, back to the 1940s [4]. Since then, this field had been developed very slowly until the 1990s, when the application of the new generation of multiple-collector magnetic-sector inductively coupled mass spectrometry (MC-ICP-MS) allowed for more precise isotopic analysis [5,6,7]. Though the measurement precision reported in early studies (for example, by thermal ionization mass spectrometry (TIMS)) was relatively low [4], the attempts of this pilot work prompted the following studies on Cu isotopes. Since last decade, researchers have devoted themselves to increasing the accuracy of Cu isotope measurement, including sample inlet system improvement, efficient Cu separation from various materials and mass bias correction [6,7,8,9,10,11,12,13,14]. As a pioneer work during this period, Maréchal et al. [6] analyzed Cu isotope composition by plasma-source mass spectrometry combined with a purification protocol to extract Cu from natural samples. Based on the recent advances in mass spectrometry and separation techniques, Cu stable isotopes could be accurately determined, with the current 2SD precision better than 0.05‰ [10,13,15], and its application has been largely extended to variable surface environments even related human health. Hitherto, a large number of studies regarding theoretical, experimental and application data sets of Cu isotope compositions have been published. These studies have largely improved our knowledge on Cu biogeochemical behaviors in biosphere, hydrosphere and lithosphere, and to different extent on Cu isotopic fractionation mechanisms in various geochemical, biological and metabolism processes [15,16,17,18,19,20,21].

Preceded this work, Albarède has reviewed some earlier papers about the isotope geochemistry of Cu (and Zn), with a focus on the analytical techniques and Cu isotope variations in biological and geological samples [8]. It must be emphasized that, in the last decade, a large number of papers has been published, which considerably enlarged our knowledge on Cu and its isotopes, especially on the mechanisms that fractionate Cu isotopes in variable processes. Most recently, Moynier et al. [22] reviewed the isotope geochemistry of Zn and Cu, such as both isotopic signatures in natural samples (even extra-terrestrial materials), their fractionation factors and experimental controlled mechanisms. Different from the previous contributions, we give here a tentative review of the recent publications on Cu isotopes, with a main focus on Cu isotope systematics in variable surface repositories and in animal and human organisms, and Cu isotopic fractionation mechanisms encountered in different surface biogeochemical and metabolism processes. The main goal of this review is to show the potential of Cu isotopes in studying the behaviors of Cu during variable environmental processes, and the linkage of Cu presence to the relative health effects. 

## 2. Analytical Methodologies

### 2.1. Cu Separation from Sample Matrix

Before isotope determination by MC-ICP-MS, Cu should be extracted and purified from sample matrix, especially from Zn, Co and Cr that might interfere Cu isotopes during instrumental analysis. Practically, different anion-exchange resins were examined and used to purify Cu from natural samples, including AG1×2 (100–200 mesh, chloride form), AG1×4 (100–200 mesh, chloride form), AG 1×8 (BioRad, 100–200 mesh and 200–400 mesh), AG 1×10 (BioRad, 100–200 mesh), and AG MP-1 (100–200 mesh, chloride form) [6,11,23,24]. In these studies, efforts were made to guarantee: (1) high purity; and (2) high recovery of Cu in the final eluant during extraction and separation of this element from sample matrix, because the unsatisfactory recovery would lead to an unexpected fractionation of Cu isotopes. In a pioneering work, Koster used first AG 1×8 resin to extract Cu from silicate rock samples, but failed to recover all Cu [23]. Over a decade after, Walt et al. [24] found that anionic exchange resin AG MP-1 (100–200 mesh, chloride form) could isolate Cu(II) more efficiently, and then in 1999, Marechal et al. [6] applied this resin to purify Cu from various natural samples including silicates, carbonates and organisms. Larner et al. [11] established a new Cu extraction technique prior to the isotope analysis using AG 1×8 (BioRad, 200–400 mesh). In their method, univalent Cu^+^ was oxidized to Cu^2+^ on the resin and then Cu^2+^ was extracted into the elution, which allowed for a higher recovery (100 ± 2%) and lower procedure blanks. Since the ocean plays an important role in the global biogeochemical cycle of Cu, two separation protocols were developed for purifying trace Cu from seawater [25,26]. Bermin et al. [25] firstly measured Cu isotopes in seawater, and demonstrated that both chelation and Mg(OH)_2_ coprecipitation followed by anion exchange chromatography can efficiently separate and purify Cu from seawater samples (recovery of about 99 ± 9%). Later, in 2013, Takano et al. [26] developed another protocol composed of two chromatographic columns (Nobias-chelate PA1 resin and AGMP-1 (100–200 mesh, Bio-Rad, Berkeley, CA, USA)), which significantly facilitated the accurate analysis of seawater Cu isotope ratio.

### 2.2. Cu Isotope Analysis

Nowadays, Cu isotope determinations are generally performed on MC-ICP-MS (Nu instruments, UK or Neptune, Thermo-Fisher, Bremen, Germany). The Cu isotope data for a sample are expressed as δ^65^Cu (‰) notation, with a comparison to the ^65^Cu/^63^Cu ratio of the Cu standard solution, calculated by the formula:

δ^65^Cu = [(^65^Cu/^63^Cu)_sample_/(^65^Cu/^63^Cu)_standard_ − 1] × 1000 (1)

where standard represents the international reference material NIST SRM 976 [6]. Recently, researchers have made efforts to improve the measurement precision, in different analytical aspects such as Cu separation from sample matrix [6,11,23,24], highly purified Cu standard development [10,13,27,28], and mass discrimination correction [10,29,30,31].

The NIST SRM 976 Cu solution was the commonly recommended standard for Cu isotopic analysis and widely used to test the instrument stability. Unfortunately, the NIST SRM 976 is no longer available; an alternative reference material is thus needed to replace this standard. This additional standard should be pure and sufficient to meet the global need for long term investigations on Cu isotopes and be (inter-) calibrated against NIST SRM 976. This also helps for testing and verifying accurate Cu isotope analysis [10,13,27]. Researchers are actively engaged in this job. For example, the Romil Cu standard (>99.999%, an industrial single element standard, Romil Ltd., Cambridge, UK) was calibrated relative to NIST SRM 976 and used for isotopic analysis [7,11,32,33]. Other standards such as ERM-AE633 and ERM-AE647 from the Institute for Reference Materials and Measurements (IRMM, Belgium), GSB Cu from Chinese National Reference Material (>99.99%), the National Standard Substances (China), and NWU-Cu-A and NWU-Cu-B (China) were also suggested and displayed δ^65^Cu values of +0.01 ± 0.05‰, +0.21 ± 0.05‰, +0.44 ± 0.04‰, +0.91 ± 0.03‰ and δ^65^Cu = −0.05 ± 0.03‰ relative to the NIST SRM 976 [13,27], respectively. Though commercially available, the different batches of one single standard may possess various isotopic ratios [7,8]. Thus, the reference material selected for the future should be calibrated and compared by different laboratories worldwide. 

### 2.3. Mass Bias Correction

Since instrumental fractionation may eventually be produced in MC-ICP-MS measurements, the raw data (instrumental mass bias) must be corrected to obtain reliable isotope ratio [10,28]. Normally, mass bias could be corrected in several ways: (1) sample-standard bracketing (SSB), in which one unknown sample was determined between two standard analyses; (2) external normalization, by measuring an element (dopant) of similar mass (and/or chemical properties) with the analyte added in the sample; (3) a combination of external normalization and sample-standard bracketing, or (4) double-spike [34,35,36]. Because Cu has only two isotopes, double-spike is not applicable. While the other three methods were normally used, Zn was generally chosen as a dopant for mass bias correction in Cu isotopic analysis [14,31,37], and vice versa [6,34]. In addition, Ni and Ga were alternative candidates for such issue [10,38]. To be mentioned, the concentration ratio (and matrix) of dopant/Cu in the samples should be identical to that in the standard in order not to induce additional analysis bias [10].

## 3. Cu Isotopic Fractionation during Low Temperature Processes Revealed by Experimental Studies

**Figure 1:**
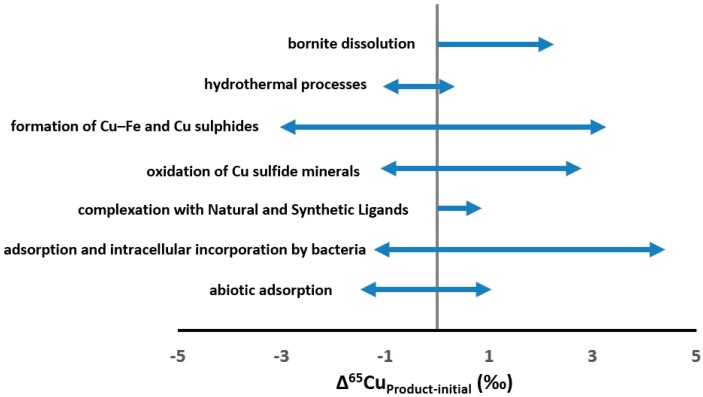
Fractionation(Δ^65^Cu) of Cu isotopesin low temperature processes [37,38,39,40,42,43,44,45,47,48,50,52,53,54,56,57].

Many experiments have been conducted in laboratory to better understand the Cu isotopic fractionation mechanisms in potential migration and transformation processes involved in the surface biogeochemical cycling of Cu. Previous studies showed that Cu isotope fractionation could be induced by various low temperature processes, such as adsorption, mineral dissolution, redox reaction, complexation with minerals and/or organic matters, and biotic incorporation [38,39,40,41,42,43,44,45,46,47]. In these processes, the isotopic fractionation (expressed as Δ^65^Cu_P-I_) was determined for describing the enrichment or depletion of Cu isotope (^63^Cu or ^65^Cu) in the product compared to the initial materials, and was calculated as following:

Δ^65^Cu_P-I_ = δ^65^Cu_P_ − δ^65^Cu_I_ (2)

where δ^65^Cu_p_ and δ^65^Cu_I_ were the isotope ratio in the product P and initial material I, respectively. Figure 1 summarizes Δ^65^Cu (−1.46 ~ +4.40‰) in low temperature processes. Studies on leaching of different ores or minerals indicated that, in general, heavy Cu isotope (^65^Cu) was preferentially released into the acidic solutions (with Δ^65^Cu_solution-solid_ as large as +3‰), while the lighter remained in the mineral or residual phases [39,42,47,48,49,50,51]. The pH might influence the Cu isotopic fractionation to different degrees depending on the geochemical reactions. For example, the pH variation impact on Cu isotopic fractionation were significant, slight and insignificant respectively during Cu interaction with soil and aquatic microorganisms (and metal oxy(hydro)oxides), adsorption onto kaolinite and complexation with insolubilized humic acid [40,50,52]. In a chalcopyrite oxidation experiment, Rodríguez et al. [45] showed that heavy Cu isotope was easily oxidized and released into solution at the beginning, and as the leaching progressed, the leachate displayed increasingly similar δ^65^Cu values to the chalcopyrite, close to an equilibrium fractionation in a closed system. Large isotopic fractionation was observed in aqueous Cu(II) reactions with CuS and FeS with heavy isotope (^65^Cu) enriched in solution (Δ^65^Cu_solution-mineral_ ~ +3‰) [39,53]. Complextion could produce Cu isotope fractionation with an enrichment of heavy Cu isotope in the organo-Cu complexes (Δ^65^Cu_complex-free_: +0.14 to +0.84‰) [38,40]. During adsorption processes, heavy Cu isotopes could be preferentially adsorbed to Fe and Al oxy(hydr)oxides or bacteria as well thus induced an isotopic offset of Δ^65^Cu_solution-solid_ [43,50,54,55]. In fact, the distribution amongst different species would also induce large Cu isotope fractionation, as calculated by Fujii et al. [37] that Cu in dissolved carbonates and sulfates was isotopically heavier than free Cu.

Cu isotopic fractionation may also be triggered by biological processes. It is worth mentioning that Mathur et al. [43] observed distinct enrichment of δ^65^Cu by up to +3.0‰ in Cu sink on bacteria surfaces in contrast to the aqueous phase. Navarrete et al. [44] performed a further surface adsorption research and indicated that the apparent separation factors (Δ^65^Cu_solution–solid_) produced by live bacterial cells ranging from +0.20 to +2.60‰, and live-bacteria intracellular incorporation preferred the lighter Cu isotope even produced a significant larger fractionation of Cu isotopes (apparent Δ^65^Cu_solution-solid_ ranging from ~+1.0 to +4.4‰). They suggested that the cell enrichment of the light Cu isotope was probably a metabolically-driven phenomenon rather than caused by reversible surface adsorption, a result consistent with the previous study [58]. 

## 4. Cu Isotope Compositions in Earth Surface Reservoirs

### 4.1. Solid Reservoirs on the Earth Surface

#### 4.1.1. Soil Weathering Profiles

The transportation and bioavailability of Cu in soils were strongly controlled by the chemical Cu forms and affected by water-induced alterations under variable soil redox conditions. Cu could exist in three forms as elemental Cu, Cu^+^ and Cu^2+^ in soils and Cu^2+^ was the major form [59,60]. Several studies reported Cu isotopic fractionation and the vertical variation in soils, and showed diverse variation tendencies for different types of soil [59,61,62,63]. Bigalke et al. [62] studied the hydromorphic soils and found an interesting correlation of δ^65^Cu with Fe oxide proportion at different crystallization stage, and suggested an important effect of redox condition (caused by water saturation) on Cu isotope variation. This was further confirmed by the sequential chemical extraction study of the same group on a soil core from a freshwater intertidal mudflat, which showed δ^65^Cu values increased with depth while the redox conditions changed from oxic to strongly anoxic conditions [61]. Recently, Kusonwiriyawong et al. [64] studied the Cu isotope variations related to the chemical partitioning changes by leaching the soils with different reactants (F1–F5:NH_4_NO_3_-extractable, NaOAc-extractable, NH_4_O_x_-extractable, hot H_2_O_2_/NH_4_OAc-extractable and residual fractions, respectively). They observed a strong effect of redox conditions on Cu partitioning and isotope composition in soils and a Cu availability declining under anoxic conditions. In addition, Cu isotope ratios might display seasonal variations with organic matter and redox condition concomitantly changes [61]. Bigalke et al. [59] also found that, unlike the hydromorphic soils, oxic weathering without evident podzolization induced an increasingly light tendency of δ^65^Cu values with depth. 

Other studies reported also large variations of Cu isotope ratio during surface weathering of shales. Black shales, one of the main Cu pools that can release Cu to water systems, displayed large isotope variation (between −6.42 and +19.73‰), with heavy Cu isotope significantly preferred to release into fluids, which was in line with leaching experimental results [65]. For instance, the soils were found depleted in ^65^Cu (average δ^65^Cu = −0.5 ± 0.2‰) during shale weathering compared to the parent rocks (average δ^65^Cu = +0.03 ± 0.15‰), while the pore waters were enriched in ^65^Cu (average δ^65^Cu = +1.14 ± 0.44‰) [66].

#### 4.1.2. Lithosphere

**Figure 2:**
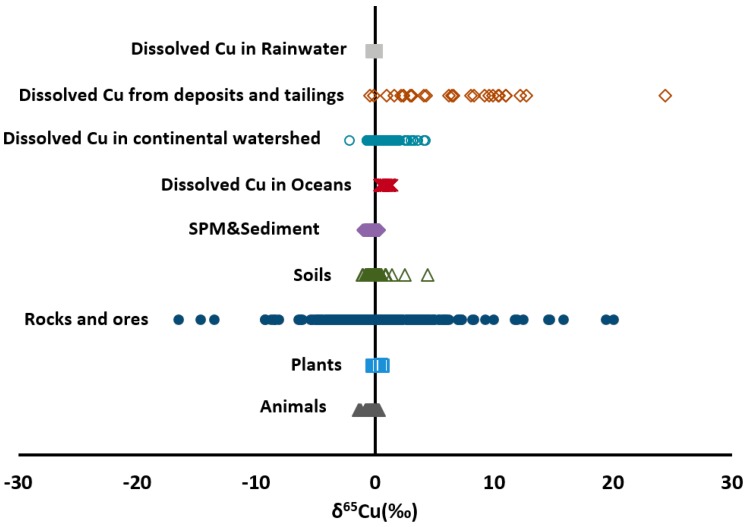
The δ^65^Cu (‰) values of various natural materials [6,7,15,19,20,21,26,32,40,59,61,63,64,65,66,68,70,72,73,76,80,81,82,83,84,85,86,87,88,89,90,91,92,93].

Lithosphere is an important Cu reservoir on the earth. About 75% of the world’s copper are supplied by porphyry copper systems [67]. There are three major mineral categories according to Cu isotopic distribution: leached, supergene and hypogenezone [68]. Large variation of δ^65^Cu (−16.49 to +20.04‰) was observed in ores and minerals [6,59,69,70], averaging +0.11‰, as summarized in Figure 2. Hypogene minerals (e.g., chalcopyrite, chalcocite, covellite, and pyrite enargite) usually had a narrow δ^65^Cu variation around zero, whereas leached (e.g., hematite, jarosite, and goethite) was isotopically lighter and supergene minerals (e.g., chalcocite, hematite, and Cu oxide) isotopically heavier [57,68,71,72,73,74]. Narrow variations of Cu isotopic composition in silicate reservoirs (e.g., basalts, granites) were reported recently [75,76]. It suggested that high-temperature magmatic processes do not induce large Cu isotope fractionation [30,72,77,78,79]. In addition, Liu et al. [75] found the metasomatized peridotites displayed relatively wider ranges of δ^65^Cu values (−0.64 to +1.82‰) than non-metasomatized peridotites (from −0.15 to +0.18‰), indicating metasomatism might trigger significant isotopic fractionation.

Cu isotope can be significantly fractionated (~36‰) in low-temperature ore deposits, and this was mainly attributed to the processes such as redox reactions, mineral dissolution and precipitation [14,39,47,48,49,53,66]. Zhu et al. [7] found that Cu formed at low temperature displayed large δ^65^Cu variations in native Cu, malachite, azurite and chalcopyrite at a single site, suggesting that the δ^65^Cu variations were mainly caused by low temperature aqueous process fractionation rather than source heterogeneity. In the sedimentary mineralization, the Cu isotopic ratio of Cu sulphide concretions exhibited abimodal distribution (δ^65^Cu = −0.91 ± 0.51‰ and −3.32 ± 0.29‰), and redox and salinity variations were likely the major regional controlling parameters affecting the Cu isotopic ratio [90]. Interestingly, Wilson et al. [21] measured Cu isotopes with a goal of identifying their origin of mislabeled Cu minerals. Since the δ^65^Cu (+5.63‰) of their Cu crystal specimen was significantly different from Michigan Cu samples with a tight cluster of Cu isotope ratio (averaging δ^65^Cu ~ +0.30‰), but in accordance with the Namibian chalcopyrite (δ^65^Cu: +1.73~ +9.27‰), signifying a possible origin of these minerals from Namibia.

### 4.2. Biosphere

As a redox-active metal, Cu functions as an enzyme-activator and is a significant prosthetic-group-part of many enzymes [94]. Given the importance of Cu in biological activities, researchers have carried out a large number of studies on Cu isotopic fractionation and relative mechanisms during soil-plant uptake and within-plant Cu translocation [59,95,96]. Recent studies indicated that plants systematically enriched in lighter Cu isotope compared with the soil in which they grew, and reduction was the major ubiquitous uptake mechanism. Free Cu(II) was reduced to Cu(I) at the surface of the plasma membrane by specific reductase and then Cu(I) was transported into plant roots by a transporter protein (COPT1) with an enrichment of isotopically light Cu [18,95,97,98]. Various within-plant Cu isotopic fractionation direction and magnitude were observed among different plant organs (root and stem), different plant tissues (xylem and phloem), and plant senescence caused remobilization [18]. Moreover, different plant components also had different Cu isotope compositions, for example, in the dicot, lentil (*Lens culinaris*), and two monocots, virginia wild rye (*Elymusvirginicus*) and hairy-leavedsedge (*Carexhirsutella*), the shoots were isotopically lighter than the germinated seeds and the Cu isotopic signature of leaves had a correlation with the plant heights [98]. Oat plants exhibited no evident fractionation during translocation, but heavier Cu isotope was preferentially transported to shoots in tomato, and the isotopic variations within different tissues were mainly due to redox cycling [97]. In addition, the magnitude of soil-plant fractionation was found to be impacted by free Cu ion species (modulated by the reduction process) in soil solution and to be correlated with soil pH values [18]. 

**Figure 3:**
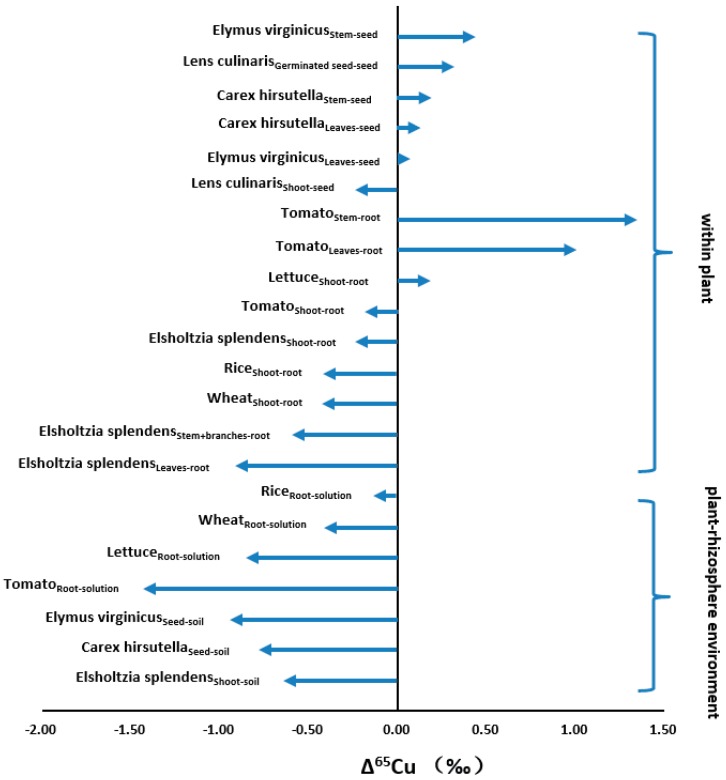
Cu isotopic fractionation factors (Δ^65^Cu) during soil-plant uptake and within-plant processes [18,84,95,97].

Summary on fractionation factors (Δ^65^Cu) during plant–soil and within-plant processes are illustrated in Figure 3. As was shown, Δ^65^Cu variation between plant organs and rhizosphere environment (soil/solution) are −1.43 to −0.11‰, while the in-plant Δ^65^Cu ranged from −0.90 to +1.35‰. The largest variation of Δ^65^Cu_plant-rhizosphere environment_ (−1.43‰) was observed between root and solution in tomato, while the maximum in-plant Δ^65^Cu (+1.35‰) was found between stem and root of tomato.

### 4.3. Hydrosphere

Studies carried out in last decade have shown that Cu isotope approach could provide new insights into fingerprinting sources and processes (and mechanisms) that Cu involved in various aquatic environments, including rivers, streams and oceans [19,34,83,84,85,87,89,99,100]. According to the literature data, dissolved δ^65^Cu in rivers ranged from −0.69 to +1.55‰, with the average +0.53‰ ± 2SD; and the suspended particulate matter (SPM) in aquatic environment (wetland, estuaries and rivers) displayed a complementary light pool (−1.02 to +0.09‰, averaging −0.31‰ ± 2SD) [85,88,89,99]. Generally, dissolved Cu in the aqueous repertories was isotopically heavier than the solid Earth such as SPM [89], however, rivers displayed different trend significantly impacted by anthropogenic sources with the lightisotope enriched in waters [85]. For example, in a small Mediterranean vineyard river, dissolved Cu displayed positive δ^65^Cu (+0.31‰), similar to the SPM (δ^65^Cu = +0.26‰), and higher than the local bedrock (+0.07‰), due to the significant contribution from long-term application of coppery fungicides [88]. The δ^65^Cu values of the surface water were found increasingly lighter along the distance from the Pebble Porphyry Cu-Au-Mo deposit, indicating a gradually varied contribution from high-temperature-formed mineral weathering [87]. 

Up to now, the fractionation of Cu isotopes triggered by in-river processes (adsorption, desorption, biological processes, etc.) has rarely been reported. Ilina et al. [99] showed that the dissolved and the colloidal Cu in Palojoki River displayed similar δ^65^Cu values, and attributed this to the high isotopic exchange rate between the colloidal and ionic Cu. Another interesting study on wetland showed that the dissolved Cu was depleted in ^65^Cu when traversing the wetland (Δ^65^Cu_inlet−outlet_ between +0.03 and +0.77‰), probably caused by Cu adsorption onto organic matters and aluminum minerals [84].

Cu isotopes have also been used to trace the impact of mining or smelting activities on aquatic systems. Sulfide-bearing tailing profiles showed that light Cu isotope was enriched in the oxidized zone than in the original materials [84]. Song et al. [73] observed a large δ^65^Cu variation (−0.44‰ to +24.4‰) in stream water (impacted by mining activities at the Dexing Mine, the largest Cu mine in Asia), which could be stemmed from pyrite tailings and chalcopyrite weathering. Both results were consistent with the leaching experiments that heavy Cu was preferentially enriched in solutions [42,44,45,48]. Thapalia et al. [20] performed a research on Cu isotope application to trace the sources and history of the Cu deposition in a sediment core from Lake Ballinger adjacent to Seattle, Washington, USA, indicating mean δ^65^Cu values possibly influenced by the atmospheric deposition of smelter exhaust is about +0.94 ± 0.1‰ (2σ, n = 6) between 1900 and 1979.

Except for continental aquatic environments, researchers also conducted studies on Cu isotope compositions in sea water sampled from the English Channel, Atlantic, Pacific, Indian oceans, andarctic gyre and determined a large δ^65^Cu variation of +0.38 to +1.44‰, averaging +0.70‰ [19,25,83,89]. In general, the seawater exhibited heavier Cu isotopic ratio than the riverine input (−0.69 to +1.55‰, averaging +0.53‰). This indicated that the heavy isotopic signature of seawater was likely produced by intra-oceanic processes (with light isotope preferential scavenging into sediments), rather than continental input [19,85,89]. In addition to the consistent scavenging effect that increase/decrease ^65^Cu in deep seawater, Takano et al. [19] also demonstrated that Cu isotopic composition of the surface seawater (from the North and SouthAtlantic, North Pacific and South Indian oceans) was deviated from the mixing of deep seawater, precipitation and river, and thus could be induced bybiological processes. Little et al. [31] compared Cu isotopic composition in Fe-Mn crusts to the dissolved load from the ocean basins, and showed relatively lower δ^65^Cu (+0.44 ± 0.23‰) than the bulk isotopic ratios (δ^65^Cu ~ +0.90‰) of dissolved Cu in seawater, comparable to other studies [19,89].

### 4.4. Atmosphere

Up to now, only few studies reported Cu isotopes in the atmosphere, which could however be an important tracer to trace atmospheric Cu contamination and transportation. The reported δ^65^Cu variation of the atmospheric precipitation (including snow, rime and rainwater) was −1.44 ~ +0.40‰, averaging −0.12‰ [19,82]. More studies are expected to map the regional and global atmospheric Cu isotope signatures and to investigate the mechanisms fractionating Cu isotopes in atmospheric processes.

## 5. Animals and Human Beings

Recently, isotope geochemists have made efforts to investigate metal (metalloid) isotopes in organisms of animals and human beings and to link isotopic fractionations to the metabolism processes and thus relative health effects. Significantly, Cu is a micronutrient, a catalytic and structural cofactor for various important enzymes referred to tumor development, and also behave as a modulator of inflammation. It is worth mentioning that Cu isotopic signatures have been reported in organs and body fluids of mammals including human beings, though data from human material were very limited for ethical reasons. Oxidized cations such as Cu(II) and low coordination numbers were found to favor heavy isotopes contrast to the reduced product (Cu(I)) and high coordination numbers [16]. For the aquatic animal, such as fish, Komjarova et al. [101] determined isotope ratios of Cd and Cu in zebrafish gills and found that Cd uptake was evidently restrained by Cu coexistence, suggesting an interaction between Cu and Cd, yet Cu uptake was not interfered by Cd. For terrestrial animals like sheep and mice, the δ^65^Cu values (inliver, kidney and brain) varied significantly from −1.5‰ to +1.5‰, and it was implied that Cu isotope signature could potentially be modified by the prion protein expression [80,102]. Moreover, in the mammal trophic chains, ^65^Cu was enriched by about 1.0‰ in bones of carnivores than those of herbivores [103]. 

Cu isotopic ratio has been investigated in materials from human bodies both for healthy people and cancer patients (Table 1). Cu was translocated by serum albumin, and excess Cu could be stored in metallothione as well [16]. Commonly, Cu was more concentrated in erythrocytes compared with serum. Van Heghe et al. [104] investigated Cu isotope compositions in whole blood samples from healthy volunteers, and found that Cu isotope ratios seemed to be affected by neither gender nordiet, which could be attributed to their failure in Cu extraction from samples. Albarède et al. [105] analyzed Cu isotope ratios of whole blood, serum, and red blood cells of about 50 young blood donors, and for healthy individuals, the Cu isotopic composition variationin human blood was about +1.6‰, with the lowest δ^65^Cu value in serum (−0.7‰) and the highest in erythrocytes (+0.9‰); Blood of men and women displayed visibly different Cu isotope compositions and with 0.2‰ lighter for women. More interestingly, B-type men erythrocytes could fractionate Cu to a larger extent than all the other blood types, and light Cu was mainly enriched in blood and lymphatic circulation rather than in erythrocytes [105]. Jaouen et al. [81] found that δ^65^Cu values in human blood decreased with age, and Cu isotope ratios in the Yakut’s blood were significantly lighter than those in blood of European and Japanese populations. In an archeological human bone study, the male’s bones exhibited heavier Cu isotopes than the females’ bones, implying Cu isotopes could be used as a valid tool to distinguish sexes of incomplete human remains [103].

In addition to that of healthy individuals, Cu isotopic characteristics were also studied for cancer patients. Significantly lower δ^65^Cu values were observed in the serum of patients with breast, liver, and colon cancer in contrast to those in samples from healthy individuals [16]. In the blood of hepatocellular carcinoma (HCC) patients, Cu was ^63^Cu-enriched by about 0.4‰ compared with that of the control group, and more concentrated Cu was observed in red blood cells and serum of HCC patients [106]. For colorectal and breast cancer patients, the δ^65^Cu decreased in the serum while increased in cancer cells, which was likely attributed to the extensive oxidative chelation of Cu by cytosolic lactate, and most patients in their study with serum δ^65^Cu below the threshold level (−0.35‰) did not survive [107]. Particularly, as a marker, the δ^65^Cu decline preceded biomarkers for several months, thus suggested a strong potential of Cu isotope composition as a new diagnostic tool at least for breast and colorectal cancers. 

## 6. Conclusions

Cu isotopes are increasingly studied and applied in surface biogeochemistry in recent years. As a main Cu reservoir on the earth, the lithosphere displays a large variation of δ^65^Cu (−16.49 to +20.04‰), which might result from many processes including especially the redox reaction. The surface weathering process (e.g., water–rock interaction) would modify the initial Cu isotopic signature of bedrocks, by, for example, enriching heavy Cu isotope (^65^Cu) in water, and thus light Cu isotope (^63^Cu) preferentially remains in the parent materials. The input into the ocean through continental rivers is thus generally characterized by higher Cu isotope composition. However, the anthropogenic contribution may neutralize this Cu isotopic signature by adding Cu with lower δ^65^Cu value. Though the riverine input (also atmospheric precipitation) could impact the Cu isotopic compositionin offshore seawater, the biological processes may play an important role on the budget of Cu and its isotopes in pelagic water of the open sea. Interestingly, large variation of δ^65^Cu (−1.45~+1.24‰) has been observed in animal and human materials, implying that the diet intake and metabolism processes would also induce Cu isotope variation. Importantly, several studies reported that healthy individuals and cancer patients possess different Cu isotopic ratios in the same organism, demonstrating a great potential of Cu isotopes in human health studies especially as a new diagnostic toolfor cancers. Though significant progress has been made in the last decade, more studies should be carried out on Cu isotopic fractionation in surface environmental, biogeochemical, and metabolism processes, in order to better understand the transformation of Cu (and other metals) from the environment to ecosystem and even human bodies and the relative toxicity effect of Cu (and metals).

## Figures and Tables

**Table 1:** Cu isotope composition in blood and bones of Human beings.

Material	δ^65^Cu Variation (‰)	Average δ^65^Cu (‰, 2SD)	References
Healthy women serum	−0.80~0.04	−0.28 ± 0.41	[105]
Healthy men serum	−0.64~0.06	−0.28 ± 0.40	
Healthy womenerythrocytes	−0.04~0.80	0.46 ± 0.47	
Healthy menerythrocytes	0.23~0.91	0.67 ± 0.36	
Healthy womentotal blood	−0.52~0.32	0.00 ± 0.41	
Healthy mentotal blood	−0.21~0.43	0.16 ± 0.33	
Cancer patients (HCC) serum	−0.66~0.47	−0.02 ± 0.54	[106]
Control group serum	−0.39~0.38	0.10 ± 0.45	
Cancer patients (HCC) red blood cell	−0.07~0.92	0.51 ± 0.56	
Control group red blood cell	0.57~1.24	0.88 ± 0.44	
Breast cancer patients serum	−1.45~0.12	−0.51 ± 0.52	[107]
Colorectal cancer patients serum	−0.65~0.04	−0.29 ± 0.30	
Aging men blood		0.68 ± 0.49	[108]
Control group (Young men)		0.67 ± 0.36	
Postmenopausal women		0.71 ± 0.54	
Control group (Premenopausal women)		0.43 ± 0.48	
Liver		−0.26 ± 0.22	
Vegetarian female blood	−0.75~−0.29	−0.51 ± 0.46	[104]
Vegetarian male blood	−0.22~0.23	−0.07 ± 0.52	
Omnivorous female blood	−0.14~0.17	−0.02 ± 0.34	
Omnivorous male blood	−0.28~0.09	−0.05 ± 0.41	
Russian and Yakut blood	−1.37~−0.22	−0.68 ± 0.62	[81]
Archeological women bones		−0.20 ± 0.25	[103]
Archeological men bones		−0.11 ± 0.16	
